# On the Influence of Climate, &c.

**Published:** 1830-05

**Authors:** 


### BIBLIOGRAPHICAL NOTICES.

### Dr. James Clark, *on the Influence of Climate, &c.*

But a very short time has elapsed since the first appearance of this work, and a second edition is already published. The author has carefully examined the principal places in England frequented by invalids, and he has materially improved the account formerly given of those places. The article on England, indeed, has been wholly rewritten. To the only article on the climate of the Northern Atlantic in the former edition, (that on Madeira,) Dr. Clark has in the present added a few observations on the principal islands in that ocean occasionally resorted to by invalids from Europe. A good account is also given of the West Indies. As a proof of the utility of this work, we may observe that Dr. Clark has recently received letters from Madeira and Nice, stating that the cases sent to these climates during the last season have been better selected than on any former occasion. The second edition contains nearly 100 pages more than the first.

It is needless to say that the work, in its improved state, deserves still higher praise than that we bestowed in our review of the first edition. This is one of the few medical works that will be perused with as much interest by the general as the professional reader.

